# Myocardial perfusion imaging in advanced coronary artery disease

**DOI:** 10.1111/eci.70024

**Published:** 2025-03-18

**Authors:** Roel Hoek, Pepijn A. van Diemen, Yvemarie B. O. Somsen, Ruben W. de Winter, Ruurt A. Jukema, Jorge E. Dahdal, Pieter G. Raijmakers, Roel S. Driessen, Ibrahim Danad, Paul Knaapen

**Affiliations:** ^1^ Department of Cardiology Amsterdam University Medical Center, Vrije Universiteit Amsterdam Amsterdam The Netherlands; ^2^ Departamento de Enfermedades Cardiovasculares Clínica Alemana de Santiago, Facultad de Medicina, Clínica Alemana Universidad del Desarrollo Santiago Chile; ^3^ Department of Radiology & Nuclear Medicine Amsterdam University Medical Center, Vrije Universiteit Amsterdam Amsterdam The Netherlands; ^4^ Department of Cardiology Radboud University Medical Center Nijmegen The Netherlands; ^5^ Department of Cardiology Northwest Clinics Alkmaar The Netherlands

**Keywords:** cardiac magnetic resonance imaging, chronic total occlusion, coronary artery bypass grafting, coronary artery disease, coronary microvascular dysfunction, myocardial infarction, myocardial perfusion imaging, percutaneous coronary intervention, positron emission tomography, single‐photon emission computed tomography

## Abstract

Myocardial perfusion imaging (MPI) is widely adapted as a noninvasive technique to assess the presence and extent of ischemia in patients with symptoms suggestive of obstructive coronary artery disease (CAD). However, as CAD advances, several factors can complicate the interpretation of MPI, subsequently impacting clinical decision‐making. This review focuses on the utility of MPI by means of cardiac magnetic resonance (CMR) imaging, single‐photon emission computed tomography (SPECT) and positron emission tomography (PET) in patients with advanced CAD—the latter characterized by documented CAD (i.e. prior myocardial infarction [MI] and/or percutaneous coronary intervention [PCI]), prior coronary artery bypass grafting (CABG) or the presence of a chronic total occlusion (CTO). It will discuss factors impacting the interpretation of MPI, the diagnostic performance for detecting obstructive CAD and coronary microvascular dysfunction (CMD), as well as the role of MPI in guiding revascularization.

## Graphical abstract

Myocardial perfusion imaging in advanced coronary artery disease. This review focusses on the utility of MPI by means of CMR, SPECT and PET, in patients with advanced CAD—the latter characterized by documented CAD (i.e. MI and/or PCI, prior CABG or the presence of a CTO). MPI shows acceptable sensitivity for detecting obstructive CAD but has a low specificity, questioning the incremental role of MPI as a gatekeeper for ICA. Beyond this, MPI plays a broader role by enabling CMD detection through MBF quantification. While MPI‐guided revascularization shows promise, prospective trials are needed to confirm its clinical value. Abbreviations as in Figures 1–4.

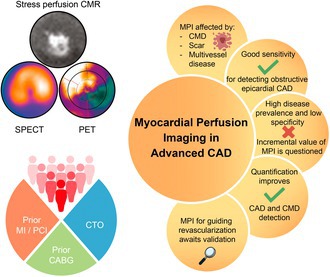

## 1 INTRODUCTION

Myocardial perfusion imaging (MPI) is a pivotal technique to discern the presence and extent of myocardial ischemia and is utilized to guide clinical decisions in patients suspected of obstructive coronary artery disease (CAD).^1^, ^2^ As of today, numerous MPI modalities are adapted by clinical practice, all of which employ modality‐specific tracers to facilitate the assessment of myocardial perfusion. In cardiac magnetic resonance imaging (CMR), gadolinium‐based contrast agents (GBCAs) are used that are visualized using serial T1‐weighted CMR images.^3^ On the other hand, single‐photon emission computed tomography (SPECT) and positron emission tomography (PET) rely on radioactive tracers.^4^, ^5^ Common tracers in SPECT perfusion imaging include Thallium‐201 or technetium‐based agents (e.g. ^99m^Tc‐sestamibi and ^99m^Tc‐tetrofosmin) whereas ^82^Rb, ^15^O H_2_O, ^13^NH_3_ and ^18^F‐Flurpiridaz are available for PET. Each of these modalities and tracers has its own unique properties, representing specific advantages as well as specific challenges.^3^, ^6^, ^7^, ^8^ These challenges can become more overt when the complexity of CAD advances. Moreover, advanced CAD might require advanced MPI techniques (e.g. absolute quantification of myocardial blood flow [MBF]), which are not clinically available for every MPI modality. In this review, we will highlight the impact of advanced CAD—defined as documented CAD (i.e. prior myocardial infarction [MI] and/or percutaneous coronary intervention [PCI]), prior coronary artery bypass grafting (CABG) or the presence of a chronic total occlusion (CTO)—on CMR, SPECT and PET perfusion imaging. We will focus on (1) complicated interpretation of MPI in these patients; (2) the use of MPI in detecting obstructive epicardial CAD and coexisting coronary microvascular dysfunction (CMD); (3) the role of MPI in guiding revascularization; and (4) future directions of MPI for use in patients with advanced CAD.

## 2 THE INTERPLAY OF MYOCARDIAL PERFUSION AND ADVANCED CAD

**FIGURE 1:**
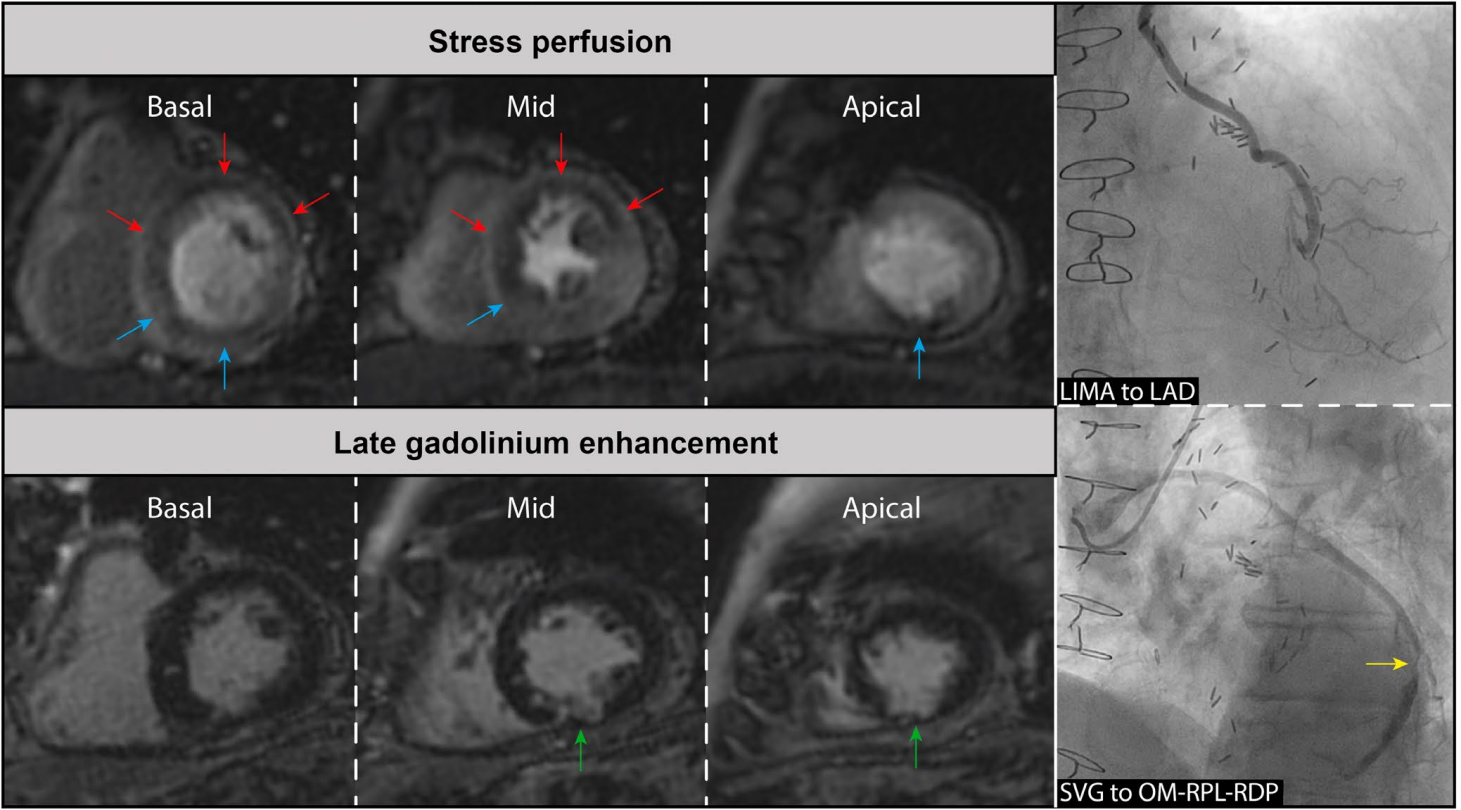
Case example of a false‐positive stress perfusion CMR in a patient with a LIMA graft. Case of a patient with prior CABG (LIMA to LAD and SVG to OM‐RPL‐RDP) who underwent adenosine stress perfusion CMR imaging because of recurrent angina symptoms. CMR showed perfusion defects in the LAD territory; red arrows, as well as the RCA territory; blue arrows. Late gadolinium enhancement imaging revealed the presence of subendocardial scar in the inferior wall (green arrows), the extent of which was smaller than the perfusion defect in this area, suggesting the presence of ischemia in the RCA territory. ICA revealed a patent LIMA to LAD and native LAD distal to the anastomosis, with retrograde filling to two diagonal branches. The SVG to OM‐RPL‐RDP had an obstructive stenosis located between the anastomosis with OM and RPL (yellow arrow). The perfusion defects in the RCA territory can be explained by this SVG lesion. However, a patent LIMA to LAD with intact runoff is observed, which does not explain the observed perfusion defect in the anterior wall of the left ventricle. This relative perfusion defect could be caused by a discrepancy between the time point of contrast arrival through the LIMA graft in comparison to native arteries and the SVG. CABG, coronary artery bypass grafting; CMR, cardiac magnetic resonance imaging; ICA, invasive coronary angiography; LAD, left anterior descending artery; LIMA, left internal mammary artery; OM, obtuse marginal artery; RCA, right coronary artery; RDP, right descending posterior artery; RPL, right posterolateral artery; SVG, saphenous vein graft.

The use of MPI is advocated worldwide for patients with a high probability of obstructive CAD.^1^, ^2^ However, in patients with advanced CAD, such as those with prior MI/PCI, prior CABG or a CTO, several factors come into play that challenge the interpretation of MPI (Table 1). First, the presence of infarcted myocardium affects tracer uptake and, consequently, MPI interpretation. While radionuclide tracers generally do not accumulate in infarcted regions, CMR uses late gadolinium enhancement (LGE) to account for scar. However, within these scarred areas, regions of viable tissue could be present.^9^ This coexistence of viable and scarred tissue within the same territory can pose a challenge for clinicians when interpreting MPI, potentially leading to misinterpretation. Second, previous MI,^10^ prior CABG^11^ and the presence of a CTO^12^ all have been associated with multivessel disease (MVD). MVD can lead to balanced ischemia, wherein there is no adequate healthy reference myocardium, which is required for accurate interpretation of MPI when relying on visual interpretation of tracer uptake images, and could cause underestimation of the extent of ischemia.^13^, ^14^ The implementation of quantitative perfusion imaging may resolve this issue.^15^, ^16^ However, for SPECT and CMR perfusion imaging, the clinical adaptation of quantitative imaging remains limited, and MVD continues to pose a challenge for these modalities. Another important factor that further complexifies the interpretation of MPI in patients with advanced CAD is the presence of CMD. Progression of CAD affects both the epicardial conduits and the microvasculature.^17^ CMD may lead to diminished myocardial perfusion and, as such, perfusion defects as seen on MPI. Indeed, CMD is an entity frequently observed in patients with advanced CAD, such as those with a prior PCI,^18^ prior MI^19^ prior CABG^20^ and the presence of a CTO.^21^ Moreover, CMD can be more prominent in infarcted myocardium in comparison to healthy myocardium, indicating that CMD can be a regional process as well.^19^ With the lack of anatomical information when performing MPI, the presence of CMD in phenotypes of advanced CAD may raise uncertainty regarding the aetiology of reduced perfusion. Importantly, in contrast to regional CMD, global CMD is a homogenous process and it may thereby elude detection by qualitative approaches. Finally, for patients with prior CABG, variations in post‐operative anatomy and the presence of competitive flow are thought to impact MPI interpretation,^22^ as well as a mismatch between the graft and native vessel size^23^ and the possibility of delayed contrast arrival through grafts (Figure 1).^24^ Overall, the interplay between myocardial perfusion and advanced CAD is complex and affected by multiple factors. In light of these complexities, the diagnosis of obstructive CAD and treatment decisions based on MPI are different from those for patients without advanced CAD.

**TABLE 1:** Factors challenging the interpretation of MPI in patients with advanced CAD.

Advanced CAD type	Cause of challenging MPI interpretation	Explanation	Challenging for which modality or type?
Documented CAD, Prior CABG and CTOs	Infarcted myocardium	Altered tracer kinetics in scarred territory. Viable tissue might be present in scarred territory.	All modalities and types
	Multivessel disease	Hinders visual interpretation. Underestimation of extent of ischemia.	Qualitative MPI
	Microvascular disease	Causes ischemia even in absence of focal obstructive stenosis. Raises uncertainty of aetiology of ischemia.	Global CMD: Quantitative modalities Regional CMD: All modalities and types
Prior CABG	Variations in post‐operative anatomy	Complicate allocation of ischemia to corresponding vessel.	All modalities and types
	Competitive flow	Complicates allocation of ischemia to corresponding vessel. Potential insufficient retrograde flow to proximal region of anastomosis, resulting in perfusion defects in patients with patent grafts without new obstructive CAD.	All modalities and types
	Mismatch graft and native vessel size	Can cause ischemia despite presence of patent grafts.	All modalities and types
	Delayed contrast arrival through grafts	Can mimic perfusion defect despite presence of patent grafts	CMR

Abbreviations: CABG, coronary artery bypass grafting; CAD, coronary artery disease; CMR, cardiac magnetic resonance imaging; CTO, chronic total occlusion; MPI, myocardial perfusion imaging.

## 3 MPI FOR DIAGNOSING CORONARY ARTERY DISEASE

### 3.1 Diagnosing obstructive epicardial disease in patients with documented CAD

Several studies report the diagnostic accuracy of different MPI modalities in patients with a history of CAD.^25^, ^26^, ^27^, ^28^, ^29^, ^30^, ^31^, ^32^, ^33^, ^34^, ^35^ The PACIFIC 2 (Functional stress imaging to predict abnormal coronary fractional flow reserve) study compared the diagnostic performance of quantitative [^15^O]H_2_O PET, qualitative stress perfusion CMR and qualitative SPECT referenced by fractional flow reserve (FFR) among patients with a history of MI and/or PCI.^25^ PET had higher sensitivity (81%) as compared to CMR (66%, *p* = .014) and SPECT (67%, *p* = .016), whereas specificity was comparable between modalities (PET: 65%, CMR: 62% and SPECT: 61%). Differences in overall accuracy did not reach statistical significance (PET: 75%, CMR: 64% and SPECT: 65%). A complete overview of the diagnostic performance of all three modalities is presented in Table 2. Interestingly, the diagnostic performance of MPI for detecting haemodynamically significant coronary stenosis in patients with documented CAD is hindered by a reduced specificity when compared to patients without documented CAD, while sensitivity is comparable.^36^ Santara et al. seem to corroborate these findings, demonstrating a low specificity of 54% using quantitative ^82^Rb PET in 53 patients (previous revascularization: 56%, previous MI: 27%), while sensitivity for detecting a stenosis ≥70% on invasive coronary angiography (ICA) was 95%.^26^ Notably, the cutoff of ischemia in this study was based on a healthy control group, which could have contributed to the large gap between sensitivity and specificity. Contrary to Santara and colleagues, the study of Esteves et al. demonstrated a specificity of 83% and sensitivity of 90% among 52 patients undergoing ^82^Rb PET again using diameter stenosis as a reference (≥70%), but employing a 4‐point relative perfusion scale for ischemia.^27^ Noteworthy, in this population, the prevalence of documented CAD was markedly lower (prior PCI 50%). Similarly to PET, studies on the diagnostic performance of CMR exclusively in patients with a history of CAD are limited to the PACIFIC 2 study. However, Merkle et al. did perform a subgroup analysis involving 169 patients suspected for progression of known CAD using ICA as a reference standard.^28^ Again, specificity was low (59%) while sensitivity was preserved (96%). Once more, this underscores the increased rate of false positive findings in patients with documented CAD when MPI is referenced by FFR or diameter stenosis, both serving as reference standards for obstructive epicardial disease. Several other studies on CMR have encompassed a population that included at least 50% of patients with a history of MI or PCI.^29^, ^31^, ^32^ These studies reported sensitivity values of 86%–96%, specificity values of 80%–84% and accuracy values of 85%–91%. Of these studies, only Manka et al.^31^ used FFR to discern the functional relevance of epicardial stenoses. Additionally, Gebker et al.^29^ evaluated only wall motion abnormalities during hyperemia rather than perfusion defects. The above‐mentioned diagnostic parameters are higher as compared to the diagnostic performance reported in the PACIFIC 2 study, prompting uncertainty as to what extent this discrepancy can be attributed to different prevalence of patients with a history of CAD. For SPECT, several studies included at least 50% of patients with documented CAD and reported sensitivity values of 62%–72% and specificity values of 46%–79%.^33^, ^34^, ^35^ A recent meta‐analysis pooling the results of these studies with the results of PACIFIC 2 found a sensitivity of 63% (95% CI 52%–73%) and specificity of 66% (95% CI 56%–76%).^37^ All of these studies used different ischemia thresholds (summed stress score ≥1,^33^ summed difference score ≥1,^25^ summed difference score ≥2^34^ or visual interpretation of perfusion defects^35^), which can complicate the generalizability of these results. Overall, MPI in patients with documented CAD as compared to patients without documented CAD generally provides acceptable sensitivity for diagnosing epicardial disease. Contrarily, a high prevalence of false positives is observed across all modalities. Presumably, this is caused by a reduced myocardial perfusion due to CMD in the absence of obstructive epicardial CAD. Moreover, this population demonstrates a relatively high disease prevalence, reported at 63% in the PACIFIC 2 study compared to 44% in a comparable population without documented CAD.^36^ Consequently, the majority of patients with documented CAD would be referred for ICA based on positive MPI results, given the high sensitivity and disease prevalence in combination with a high rate of false positive findings. This questions the role of MPI as a gatekeeper for ICA in patients with advanced CAD.

**TABLE 2:** Diagnostic performance of quantitative [^15^O]H_2_O PET, qualitative stress perfusion CMR and qualitative SPECT, as documented by the PACIFIC 2 study.^25^

% (95% CI)						
	[^15^O]H_2_O PET	CMR	*p*‐Value^a^	SPECT	*p*‐Value^b^	*p*‐Value^c^
Per‐patient						
Sensitivity	81 (72–87)	66 (56–75)	.*014*	67 (58–76)	.*016*	.999
Specificity	65 (53–76)	62 (49–74)	.999	61 (48–72)	.597	.999
PPV	80 (74–85)	75 (68–81)	.235	74 (68–80)	.183	.925
NPV	66 (57–75)	51 (43–59)	.*009*	53 (45–60)	.*011*	.832
Accuracy	75 (68–81)	64 (57–72)	.052	65 (58–72)	.030	.999
AUC	.80 (.73–.86)	.67 (.59–.74)	.*011*	.66 (.58–.73)	.*001*	.895
Per‐vessel						
Sensitivity	73 (66–79)	44 (35–52)	*<.001*	60 (52–67)	.*001*	.*001*
Specificity	69 (64–73)	82 (77–86)	*<.001*	70 (66–75)	.591	*<.001*
PPV	53 (49–58)	53 (46–60)	.952	49 (44–54)	.181	.358
NPV	84 (80–87)	75 (73–78)	*<.001*	78 (75–81)	.*005*	.112
Accuracy	70 (66–74)	70 (65–73)	.827	67 (63–71)	.212	.326
AUC	.76 (.72–.79)	.66 (.62–.70)	.*002*	.66 (.62–.70)	*<.001*	.937

*Note*: Bonferroni correction applied: statistically significance at *p*‐value <.0167. Significant *p*‐values are marked *in italic*. Table adapted from Driessen et al.^25^

^a^ *p*‐value between PET and CMR.

^b^ *p*‐value between PET and SPECT.

^c^ *p*‐value between CMR and SPECT.

### 3.2 Diagnosing obstructive epicardial disease in patients with prior CABG

**FIGURE 2:**
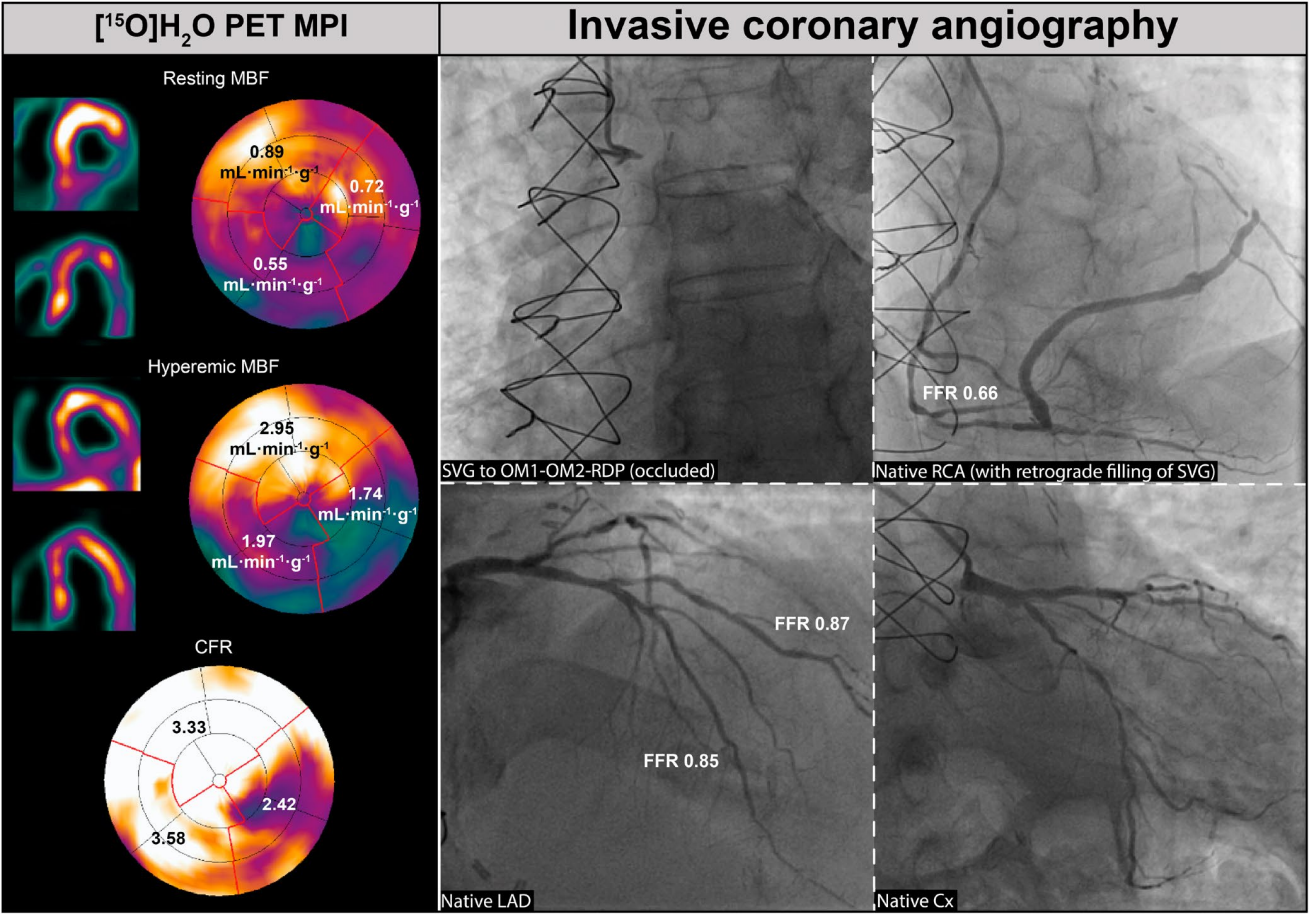
Case example of a patient with prior CABG, with perfusion defects on MPI that cannot directly be attributed to a specific lesion in a coronary artery or bypass conduit without anatomical conformation. Case of a patient with prior CABG (SVG to OM1‐OM2‐RDP) who underwent [^15^O]H_2_O PET MPI because of recurrent angina symptoms. PET MPI revealed perfusion defects in the Cx territory with hMBF of 1.74 mL·min^−1^·g^−1^ and in the RCA territory with hMBF of 1.97 mL·min^−1^·g^−1^. ICA revealed a proximally occluded SVG, a haemodynamically significant lesion in the distal RCA (FFR .66) with retrograde filling of the SVG to all anastomoses, a nonobstructed LAD (FFR .85) and large diagonal branch (FFR .87) and a significantly obstructed Cx. The perfusion defects observed on PET MPI were caused by the occlusion of the proximal SVG. Based on MPI, both RCA and Cx territories require revascularization. However, based on the anatomical information of ICA, revascularization of solely the distal RCA would be sufficient to restore blood flow to both the RCA and Cx territory, while the native Cx could be left untouched. CFR, coronary flow reserve; Cx, circumflex artery; FFR, fractional flow reserve; MBF, myocardial blood flow; MPI, myocardial perfusion imaging; PET, positron emission tomography, other abbreviations as in Figure 1.

In patients with prior CABG, recurrent symptoms related to the new onset of obstructive epicardial disease can originate from either graft failure or native disease progression.^38^ Graft failure is most frequently associated with the failure of saphenous vein grafts (SVGs), with a graft patency of up to 60% at 10 years post CABG.^39^ In contrast, the 10‐year patency of arterial grafts exceeds 90%.^39^ Native disease progression predominantly occurs in bypassed vessels, with up to 46% of bypassed native lesions progressing to a total occlusion within 5 years after CABG.^40^ Indeed, native disease progression both proximal and distal to a graft anastomosis has been linked to perfusion defects on MPI in the absence of a graft lesion.^41^ Given the lack of anatomical information derived from MPI, differentiation between graft failure and native disease progression solely based on MPI is challenging (Figure 2). The majority of prospective studies on the diagnostic performance of MPI in patients with prior CABG focused on the prediction of graft patency. These studies reported sensitivity and specificity values of 77%–80% and 69%–88% for SPECT, using both visual and semi‐quantitative interpretation,^42^, ^43^, ^44^ while a meta‐analysis by Dikkers et al. on CMR found a pooled sensitivity of 81% (95% CI 76%–86%) with a specificity of 91% (95% CI 89%–93%).^45^ Importantly, the majority of these studies comprised a mix of symptomatic and asymptomatic individuals. Prospective studies that included the prediction of native vessel disease in patients with prior CABG are solely available for CMR and reported sensitivity values of 73%–86% and specificity values of 73%–91%.^46^, ^47^, ^48^ Noteworthy, PET MPI is relatively underrepresented within this field of research. Only one study using semi‐quantitative ^82^Rb PET from 1992 demonstrated a sensitivity of 93% and specificity of 75% to detect obstructive CAD in patients with prior CABG experiencing late recurrence of symptoms.^49^ In summary, a variety of results have been reported concerning the diagnostic performance of MPI for detecting new obstructive CAD in patients with prior CABG and recurrence of symptoms. SPECT and CMR generally demonstrated moderate‐to‐good sensitivities and specificities, whereas data on PET is limited. Overall, the use of MPI as a gatekeeper for ICA seems reasonable according to the consistently acceptable sensitivities reported in all studies. Yet, in the context of the poor patency rate of SVGs and the frequently observed progression of native disease, one could argue that an initial noninvasive strategy in these patients might be redundant. However, if the suspicion of CAD new‐onset obstructive CAD is low and a direct invasive approach seems too rigorous, the use of MPI as a gatekeeper can be justified. Of note, the abovementioned studies utilized anatomical reference methods (diameter stenosis) to determine graft patency, whereas functional references are generally applied in native vessel disease. Moreover, the majority of the studies solely focused on graft patency, offering limited additional value once the decision to refer for ICA is made.

### 3.3 Diagnosing coronary microvascular dysfunction

**FIGURE 3:**
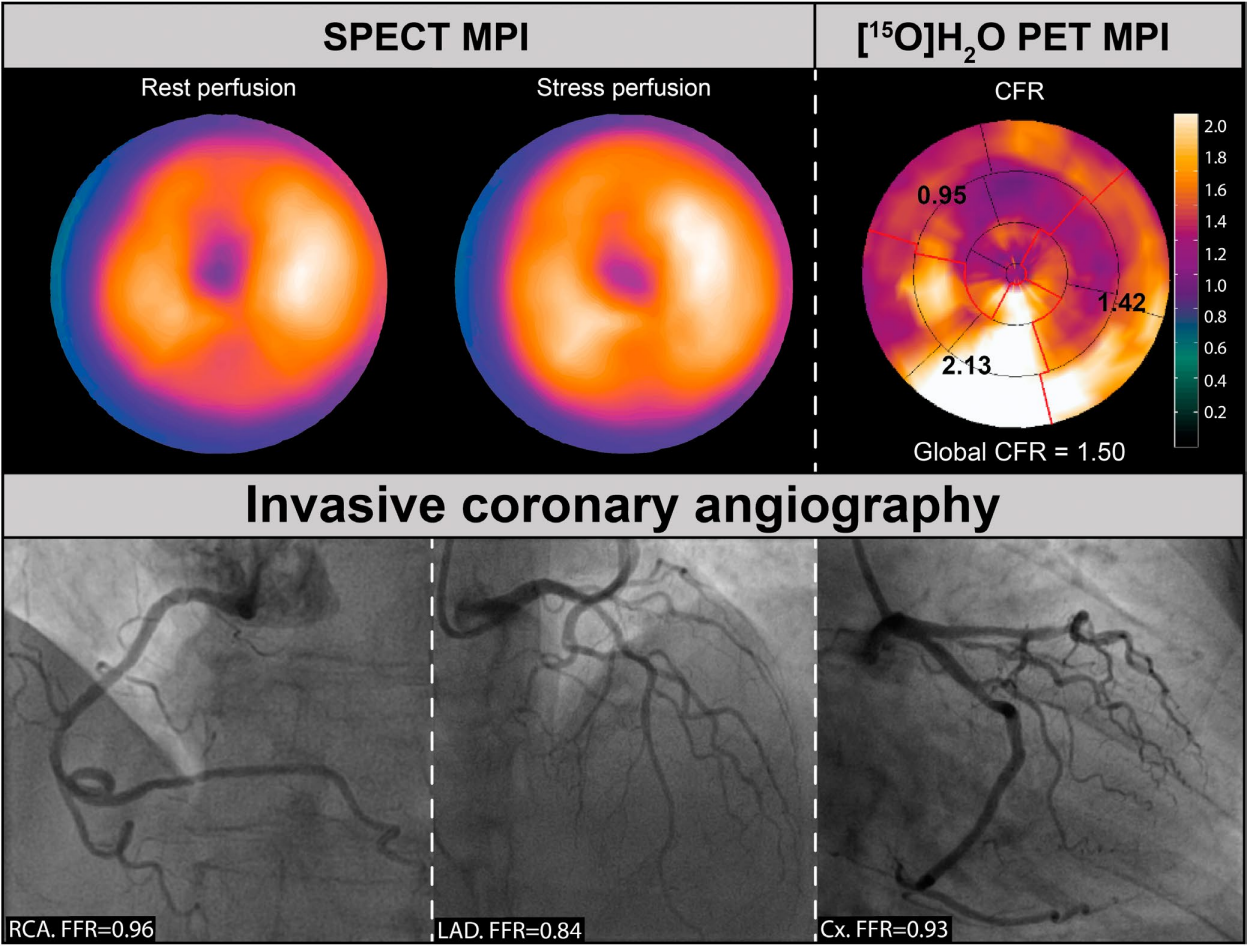
Case example of a negative SPECT and positive [^15^O]H_2_O PET in the absence of obstructive CAD. Case of a patient from the PACIFIC 2 study^25^ with prior PCI of the LAD who underwent SPECT and [^15^O]H_2_O PET MPI because of recurrent angina symptoms. SPECT MPI revealed no ischemia with a summed stress score of 0. Quantification on PET MPI revealed a vascular CFR of 2.13 in RCA territory, .95 in LAD territory and 1.42 in Cx territory, which are indicative of obstructive CAD according to the predefined CFR cutoff of ≤2.5.^85^ However, ICA revealed nonobstructive CAD in all three branches with an FFR of .96 in RCA, .84 in LAD and .93 in Cx. Global CFR of PET MPI is ≤2.0, thereby suggesting that angina symptoms in this female patient with documented CAD could be attributed to CMD, a condition that often coexists with advanced CAD. CAD, coronary artery disease; CMD, coronary microvascular dysfunction; PCI, percutaneous coronary intervention; SPECT, single‐photon emission computed tomography, other abbreviations as in Figures 1 and 2.

As previously discussed, the diagnostic performance of MPI among patients with documented CAD is hampered by a relatively high rate of false positive findings when referenced by FFR. This discrepancy between the presence of ischemia on MPI in the absence of diagnosed obstructive epicardial disease may not reflect the failure of either technique but is a mere consequence of the presence of additional factors beyond epicardial disease causing ischemia. The presence of CMD—frequently concomitant with advanced CAD—could lead to ischemia on MPI while the epicardial arteries are nonobstructive (Figure 3). Furthermore, both the loss of subtended myocardial mass following MI and increased microvascular resistance due to CMD lead to higher FFR values as compared to similar vascular territories with preserved subtended myocardium and healthy microcirculation.^50^, ^51^, ^52^ However, MPI results in patients classified as ‘false positives’ for diagnostic study purposes are not without value. In the absence of obstructive CAD, a CFR cutoff of <2.0 is universally applied for the identification of CMD across both invasive and noninvasive imaging modalities.^53^ Also, with the use of CFR and hMBF, MPI allows for the detection of the ‘endogenous type’ of CMD, in which CFR is reduced by an elevated resting MBF, while hMBF is preserved.^54^ The definition of CMD based on these numerical parameters underscores the necessity of quantitative MPI for noninvasive CMD detection (Figure 3). Of all noninvasive imaging modalities, PET MPI is considered the noninvasive gold standard for MBF quantification and thereby CMD detection,^55^ although quantitative perfusion CMR (QP‐CMR) has been described as an alternative.^56^ The definitive diagnosis of CMD utilizing MPI parameters can only be confirmed subsequent to the verification of the absence of obstructive coronary artery disease, and therefore MPI should always be used in conjunction with anatomical information derived from ICA or coronary computed tomography angiography (CCTA). However, current indices of CMD can also be impacted by the presence of hidden or overt, diffuse or focal nonobstructive epicardial disease. This phenomenon would be even more pronounced in patients with advanced CAD, who generally have more of the traditional risk factors for both (non‐) obstructive CAD and CMD. To differentiate between epicardial CAD, CMD or the combination of both, De Bruyne et al. proposed the concept of microvascular resistance reserve (MRR) as a descriptor of the microcirculatory function irrespective of the presence of epicardial disease.^57^ In summary, MPI in conjunction with epicardial anatomical information can be valuable for the detection of CMD. Moreover, the impact of epicardial CAD on current indices of CMD could obscure the true status of the microvasculature, a challenge possibly addressed by MRR.

## 4 MPI FOR GUIDING REVASCULARIZATION

### 4.1 Guiding revascularization in documented CAD

Following the FAME 2 (Fractional Flow Reserve–Guided PCI versus Medical Therapy in Stable Coronary Disease) trial,^58^ international guidelines advocate the use of FFR for decision‐making regarding revascularization in patients with stable CAD.^1^, ^2^ Both the European and American guidelines also advise to discern the functional significance of an epicardial stenosis by invasive pressure measurements like FFR if noninvasive imaging is lacking. These statements suggest a central role for noninvasive imaging in the referral process for ICA and subsequent revascularization decision‐making. However, the ISCHEMIA (International Study of Comparative Health Effectiveness with Medical and Invasive Approaches) trial failed to demonstrate an improved outcome among patients with moderate‐to‐severe ischemia referred for ICA as compared to a conservative medical treatment.^59^ Importantly, only 19% of patients had a prior MI, and 20% a prior PCI, questioning the generalizability of the results for patients with documented CAD. Interestingly, a sub‐study of ISCHEMIA showed a reduction in the occurrence of a combined endpoint of cardiovascular death or MI among patients with reduced left ventricular ejection fraction (LVEF) or prior heart failure randomized to the invasive treatment arm compared to a conservative strategy.^60^ Patients included in this sub‐study had a markedly higher prevalence of prior MI and PCI compared to the excluded patients, which may indicate that decision‐making based on MPI could be valuable in this population. Additionally, a recent observational study assessed the relationship between quantitative ischemia and early revascularization across several contemporary populations and identified a benefit of revascularization in patients with documented CAD although at a higher ischemia threshold than patients without documented CAD.^61^ Several other retrospective studies focused on the applicability of MPI in patients with documented CAD. Patel et al. showed that revascularization of patients with an ischemic burden (IB) >10% on semi‐quantitative ^82^Rb PET significantly ameliorates angina severity and improves overall health status at 12‐month follow‐up, as compared to a conservative treatment.^62^ This is in accordance with the health‐status analysis of ISCHEMIA.^63^ For SPECT, Hachamovitch et al. demonstrated that patients with a prior revascularization had a survival benefit from revascularization when the IB on relative perfusion interpretation exceeded 10%, whereas no such benefit was present when a patient had prior MI or a scar burden >10%.^64^ Comparable to SPECT, while patients with known CAD and ischemia assessed by CMR had a survival benefit from revascularization, those with solely a history of MI did not exhibit a similar advantage.^65^ Several prospective studies on MPI‐guided revascularization are currently awaiting results. The randomized CENTURY trial (NCT00756379) will incorporate ^82^Rb PET‐derived coronary flow capacity—a parameter that integrates hMBF and CFR to assess the physiological severity of CAD—to guide revascularization versus a standard care strategy, including 43% of patients with documented CAD.^66^ Although final results are not yet available, initial findings indicate that a comprehensive treatment strategy—combining intense lifestyle modification with serial quantitative PET assessment for clinical decision‐making—can improve patient prognosis.^67^ Additionally, the iMODERN trial (NCT03298659) will randomize between iFR and stress perfusion CMR‐guided revascularization of nonculprit lesions in patients with ST‐elevation MI.^68^ To conclude, MPI‐guided revascularization may result in improved patient outcomes and symptom alleviation. However, data supporting this statement is of retrospective nature or derived from smaller sub‐analyses from large randomized control trials that are not adequately powered to address this question. As such, randomized trials focused on this specific patient population are eagerly awaited.

### 4.2 Guiding revascularization in patients with prior CABG

Revascularization of patients with a history of CABG may be necessary in cases where lesions are present in unprotected native arteries or bypass conduits. An estimated 13% of patients who have undergone CABG subsequently receive revascularization within 10 years following the initial CABG.^69^ In native vessel disease, a discrepancy is known to exist between anatomical and physiological relevance estimates of lesion severity.^70^ Notwithstanding the advice of current guidelines to guide revascularization using FFR,^1^, ^2^ revascularization of bypass conduits in patients with prior CABG is primarily driven by anatomical assessment.^71^ This disparity is driven by a scarcity of studies on the application of invasive physiologic assessment in grafts, which are of retrospective design and show conflicting results for guidance of revascularization.^72^, ^73^, ^74^, ^75^ By depending on anatomical assessment of bypass grafts, the potential impact of competitive flow from a nonoccluded native vessel and the presence of collaterals on myocardial perfusion is not considered.^76^ MPI could potentially serve as an alternative functional assessment instead of invasive physiological interrogation of bypass grafts. Abnormalities on MPI have been associated with impaired prognosis,^77^, ^78^, ^79^, ^80^, ^81^, ^82^, ^83^ although, as mentioned before, a wide array of pathophysiological processes other than obstructive epicardial disease can contribute to ischemia on MPI in patients with prior CABG. In a recent study by Vester et al., quantitative [^15^O]H_2_O PET predicted subsequent PCI at a stress MBF value of 1.36 mL·min^−1^·g^−1^.^84^ This value is remarkably lower than the FFR‐defined cutoff of 2.3 mL·min^−1^·g^−1^ commonly used in native vessel disease,^85^ thereby underscoring the difference between MPI in native vessel disease and patients with prior CABG. As stated before, a mere analysis of myocardial perfusion in patients with prior CABG without information regarding the anatomical status of the vasculature would not be sufficient to reveal the aetiology of a perfusion defect, thereby prohibiting the potential of MPI‐guided revascularization (Figure 2). A hybrid approach, integrating MPI with CCTA, has been described to address this challenge.^86^ Hitherto, there are no prospective randomized studies assessing the value of MPI for guiding revascularization in patients with prior CABG, indicating a potential domain for future research.

### 4.3 Guiding revascularization in CTOs

**FIGURE 4:**
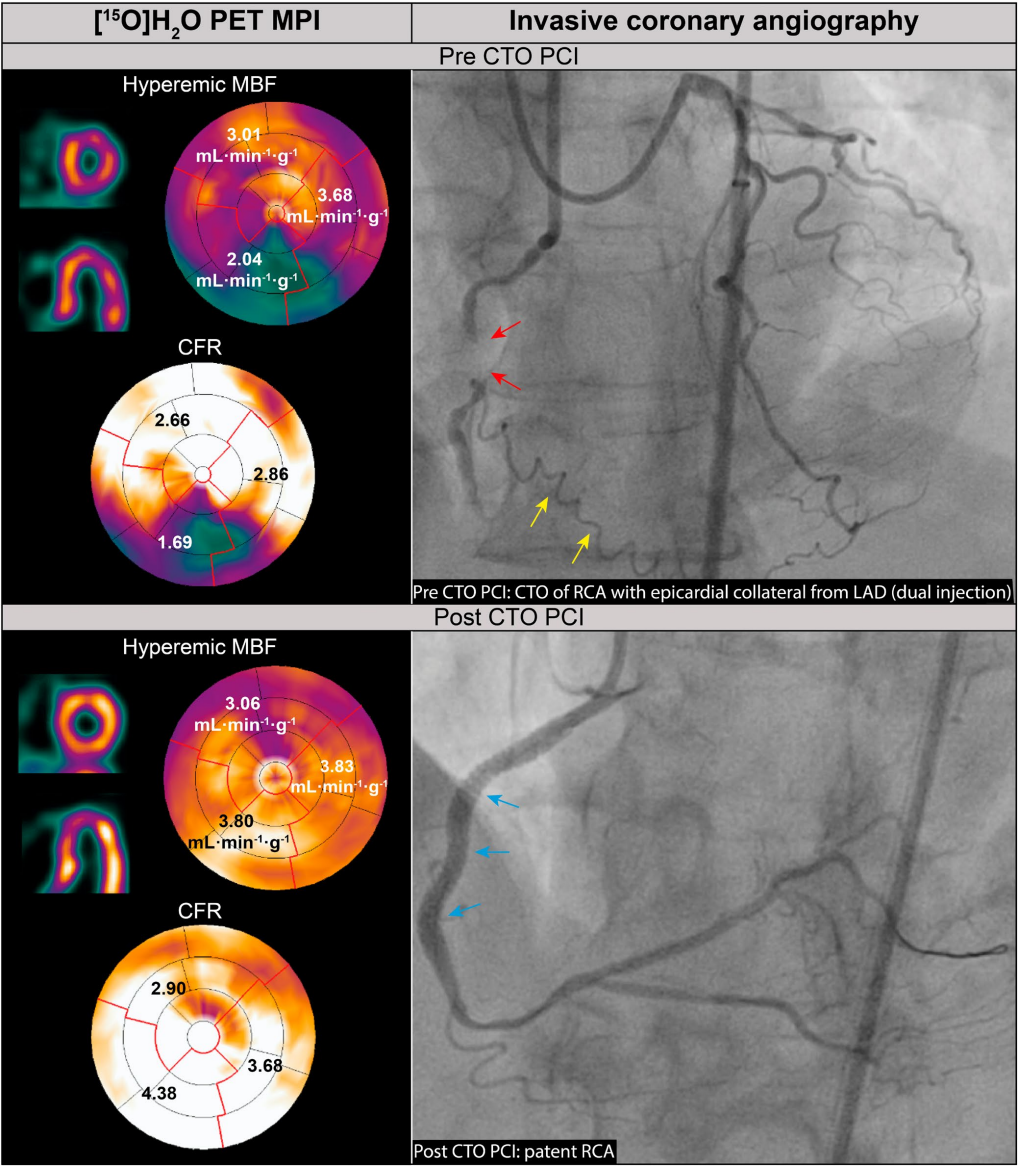
Case example of perfusion increase after PCI CTO. Case of a patient who underwent [^15^O]H_2_O PET preceding CTO PCI of the RCA. Diagnostic ICA showed a CTO of the mid RCA (red arrows) with a corresponding perfusion defect on PET (hMBF of 2.04 mL·min^−1^·g^−1^ and CFR of 1.69 in the RCA territory), despite the presence of an epicardial collateral deriving from the LAD (yellow arrows). After successful CTO PCI of the RCA (blue arrows), hMBF and CFR increased to 3.80 mL·min^−1^·g^−1^ and 4.38, respectively. CTO, chronic total occlusion, other abbreviations as in Figures 1, 2, 3.

Once the diagnosis of a CTO is established, the question arises whether or not to perform revascularization of the occluded vessel. The prospective randomized EUROCTO^87^ and DECISION‐CTO^88^ trials studied the incremental value of CTO PCI over optimal medical therapy (OMT) in terms of clinical outcomes and health status. Both trials did not show improvement in prognosis following CTO PCI. The EUROCTO trial showed an improved health status 12 months after CTO PCI as compared to OMT, whereas such a benefit was not observed in the DECISION‐CTO trial. Based on these trials, European guidelines state that CTO PCI can be performed to reduce symptom burden,^89^ whereas American guidelines are more conservative.^90^ However, these trials do not include MPI in their patient selection. The role of MPI in patients with a CTO has predominantly been studied in a retrospective manner. Schumacher et al. showed that the increase in absolute myocardial perfusion measured on quantitative [^15^O]H_2_O PET before and after PCI is equal in CTO and non‐CTO lesions (Figure 4),^91^ while de Winter et al. demonstrated an increased perfusion in remote nonrevascularized myocardial territories as well following CTO PCI.^92^ Moreover, extensive IB reduction after CTO PCI was associated with improved prognosis, whereas normalization of hyperemic MBF was related to angina relief.^93^ These findings, however, do not necessarily imply that all patients with severe ischemia on MPI benefit from revascularization, as predictors of effective ischemia reduction are not accounted for in these studies. Nevertheless, reduction of IB has been demonstrated to be more pronounced in patients with larger perfusion defect sizes,^94^, ^95^ but a concurrent comparison with symptom severity is lacking. In summary, the use of MPI for guiding revascularization in patients with a CTO has shown conflicting results. Retrospective studies suggest that CTO PCI can reduce IB, potentially influencing prognosis and symptom relief. Possibly, guiding CTO PCI with the use of MPI could improve the selection of patients who may benefit from CTO PCI. Several ongoing prospective studies aim to address this matter. The ISCHEMIA‐CTO trial (NCT03563417) plans to stratify patients with a CTO based on symptom severity and IB, and will randomize between OMT and OMT + PCI.^96^ The REVISE‐CTO Trial (NCT03756870) will randomize patients with substantial ischemia (IB > 12.5% on relative SPECT perfusion) and absence of transmural infarction (on CMR), focusing on IB reduction, cardiac function improvement and health status after 6 months.^97^ Finally, the CARISMA‐CTO study (NCT03152825) is designed to study patient‐tailored CMR scanning protocols including ischemia, viability and cardiac function testing to improve the selection of patients who could benefit from revascularization.^98^

## 5 FUTURE DIRECTIONS

Quantification of MBF has been suggested to confer a favourable diagnostic performance as compared to qualitative assessment MPI images.^15^ In this context, the severity of epicardial CAD is based on quantitative MBF using predefined cutoff values of hMBF or CFR.^85^ However, these binary cutoffs do not account for the possible presence of CMD. Interestingly, FFR is utilized to determine the presence of haemodynamically significant epicardial CAD, yet FFR was originally validated against relative flow reserve (RFR) derived from [^15^O]H_2_O PET.^99^ RFR is described as a noninvasive equivalent of the FFR and defined as the ratio between hMBF in a stenotic area to hMBF in a normally perfused area. Hypothetically, this parameter would allow patient‐tailored interpretation of quantitative MPI, as it reduces the impact of CMD on absolute perfusion measures. RFR has been studied in a population without documented CAD,^100^ but has not yet been validated in a patient population with phenotypes of advanced CAD. Quantification of MBF can be performed through CMR as well (QP‐CMR), albeit currently limited to research settings. Although QP‐CMR and visually assessed CMR show comparable diagnostic performance in patients without documented CAD,^101^, ^102^ QP‐CMR has been shown to more accurately assess the extent of ischemia in patients with MVD and could thus be of value in a population with advanced CAD.^103^ Moreover, QP‐CMR would allow different interpretation methodologies (e.g. hMBF, CFR, CFC or RFR) for CMR too. Its application has been described to frequently detect perfusion defects in patients with left internal mammary coronary artery bypass grafts.^81^ But, the first study on QP‐CMR solely in patients with documented CAD did not show an incremental value over visual CMR interpretation for the detection of haemodynamically significant CAD.^104^ For SPECT, automated analysis of relative perfusion using total perfusion deficit (TPD) has been described as an accurate tool for diagnosing significant CAD in patients without a documented history of CAD.^105^ As such, it holds promise for diagnosing CAD and guiding revascularization in a population with advanced CAD. Moreover, distinguishing between absolute and relative TPD, as recently applied in a [^15^O]H_2_O PET study, could further refine diagnosis accuracy using this method.^106^ Beyond relative perfusion, advancements in quantitative SPECT, particularly with cadmium zinc telluride (CZT)‐based detectors, have renewed interest in MBF quantification. These systems have demonstrated strong agreement with [^15^O]H_2_O PET‐derived MBF and have shown potential for diagnosing MVD.^107^, ^108^ However, as of today, studies evaluating quantitative SPECT specifically in populations with advanced CAD are lacking. A final perfusion technique that has gained momentum is computed tomography perfusion (CTP), which can be performed during the same procedure as CCTA thereby streamlining a hybrid diagnostic approach. Multiple studies found an improved diagnostic performance of CTP + CCTA over CCTA alone in patients with prior revascularization.^109^, ^110^, ^111^, ^112^ The ongoing PACIFIC 3 study (NCT04742933) was designed to further assess this modality, employing dynamic CTP in conjunction with CCTA to quantify MBF. Although the diagnostic performance of MPI in patients with advanced CAD could be further optimized by extending the possibilities of MBF quantification, its future role in this population may shift away from its current function as a gatekeeper for ICA. Of note, clinicians could continue using MPI as an initial noninvasive strategy when suspicion of obstructive CAD is uncertain and a direct invasive approach seems too rigorous. The application of MPI in patients with advanced CAD provides a more comprehensive view of a patient's vascular status beyond the scope of epicardial coronaries and can thereby contribute to patient‐tailored treatment when performed in conjunction with anatomical testing. Additionally, MPI in patients with advanced CAD could potentially identify those who would benefit from revascularization in terms of improved prognosis and symptom relief, although this needs to be confirmed through RCTs. Arguably, the future application of MPI in patients with advanced CAD should shift to complementing rather than preceding anatomical modalities.

## 6 CONCLUSIONS

The interplay between myocardial perfusion and advanced CAD is complex and affected by multiple factors. Despite these complexities, MPI generally exhibits an acceptable sensitivity to detect obstructive CAD among patients with documented CAD or prior CABG. Yet, MPI in these patients is characterized by a low specificity and a high prevalence of obstructive CAD. As such, the incremental value of MPI as an initial noninvasive gatekeeper for ICA is questioned. However, the utility of MPI in these patients is broader than merely a gatekeeper. The quantification of MBF facilitates noninvasive detection of CMD, which often accompanies advanced CAD, providing insights into the complex interplay between epicardial and microvascular disease for each individual patient. Lastly, while retrospective evidence suggests the potential of MPI‐guided revascularization in advanced CAD, the implementation of this approach into routine clinical practice awaits validation through prospective randomized trials to demonstrate its efficacy conclusively.

## AUTHOR CONTRIBUTIONS

All persons listed as authors must have contributed substantially to the design, performance, analysis or reporting of the work: Roel Hoek: main author, performed literature and case collection, drafted the manuscript. Pepijn A. van Diemen and Ibrahim Danad: provided relevant scientific input, including literature input, read and corrected the drafted manuscript and read and approved the final manuscript. Yvemarie B.O. Somsen and Ruben W. de Winter: provided relevant scientific input, aid in case collection, read and approved the final manuscript. Ruurt A. Jukema: provided relevant scientific input, including literature input, read and approved the final manuscript. Jorge E. Dahdal and Pieter G. Raijmakers: provided relevant scientific input, read and approved the final manuscript. Paul Knaapen: PI of the study group. provided relevant scientific input, read and approved the final manuscript.

## CONFLICT OF INTEREST STATEMENT

Dr. Knaapen has received research grants from Cleerly, Inc. and HeartFlow, Inc.

## Data Availability

The datasets generated during and/or analysed during the current study are available from the corresponding author on reasonable request.
